# Spent Coffee Grounds as a Source of Chlorogenic Acid

**DOI:** 10.3390/molecules30030613

**Published:** 2025-01-30

**Authors:** Krystyna Pyrzynska

**Affiliations:** Faculty of Chemistry, University of Warsaw, 02-093 Warsaw, Poland; kryspyrz@chem.uw.edu.pl

**Keywords:** chlorogenic acid, extraction, spent coffee grounds

## Abstract

Spent coffee grounds generated from the brewing of coffee cherries are rich in chlorogenic acids that are associated, to a certain extent, with a delay in the development of various chronic diseases and age-related disorders. These natural antioxidants are applied in the pharmaceutical, cosmetic, and food industries. This brief overview describes recently proposed procedures for the extraction and recovery of chlorogenic acids from spent coffee grounds, which is a low-cost and easily accessible by-product. Solvent selection and temperature control seem to be the main factors due to the thermolabile nature of these compounds. Advanced extraction technologies are generally faster and enhance extraction efficiency. Procedures for the valorization of coffee waste are the goal of a sustainable and circular bioeconomy that seeks to increase their added benefits and reduce environmental pollution.

## 1. Introduction

The requirement for an increasing amount of food produced results in the formation of more and more agro-industrial waste. Landfilling or the disposal of such waste can cause environmental pollution and economic loss [1,2,3]. However, this waste could still be a sustainable resource for several valuable phytochemicals, which can be used for the production of functional foods, pharmaceuticals, and cosmetics. Coffee, potato, and artichoke waste are especially rich in chlorogenic acids (CGAs) [4]. In addition, these compounds are also found at minor levels in mango peel and leaves, apple and sunflower by-products, tobacco waste, and citrus peels. Chlorogenic acids have been connected with several health benefits due to their antioxidant, antibacterial, antiviral, and anti-inflammatory activities [5,6]. The production of CGAs from agricultural and food processing waste represents an opportunity to create a new, economically and environmentally sustainable method, which also allows for the valorization of waste matrices.

Coffee by-products are a good example of waste valorization to recover and utilize CGAs, considering their properties. The treatment processes of the coffee beans create at least 6–8 million tons of trash every year [7]. These types of waste include several by-products, such as spent coffee grounds (SCG), pulp, parchment, silverskin, cascara, and mucilage. SCGs are the most abundant form of waste remaining after brewing coffee as a drink. About 650 kg of SCGs is obtained from one ton of coffee beans [8]. Polysaccharides of cellulose (8–15%) and hemicellulose (30–40%), with lignins at 20–30%, are the major constituents of spent coffee grounds [9,10,11,12,13]. SCGs also contain fatty acids, amino acids, proteins, lipids, phenolic compounds, caffeine, and minerals. Chlorogenic and caffeic acids are the main phenolic compounds. Some flavonoids, such as quercetin, rutin, catechin, and luteolin, have also been reported [14,15]. Genetic diversity, the maturation of beans, agricultural practices, and processing methods of coffee grains can affect the content of individual ingredients. Moreover, different conditions for coffee brewing (temperature, pressure, time, and coffee-to-water ratio) can affect the chemical composition of the final coffee brew as well as the SCGs. 

Several review papers have been published regarding the valorization of coffee by-products. Many of them described the application of coffee waste as functional foods [8,10,16,17], cosmetics [18,19,20], biofuels [21,22,23], and biomaterials [24,25]. Another group of these papers described just the valorization of spent coffee grounds, probably because it represents the most abundant form of waste [9,11,13,26,27,28,29,30]. Also, the use of other coffee by-products was discussed, such as coffee silverskin [31,32,33], pulp [34,35], and parchment [36]. 

Given its potential health benefits and several applications in different industrial sectors, chlorogenic acids have attracted considerable research interest, and SCGs may be a good source of this compound. This brief overview describes the recently proposed procedures for extracting and recovering CGAs from spent coffee grounds. Novel extraction methods such as pressurized liquid extraction, high hydrostatic pressure-assisted extraction, and supercritical fluid extraction are discussed in comparison to conventional techniques.

## 2. Chlorogenic Acids in Coffee

**Figure 1:**
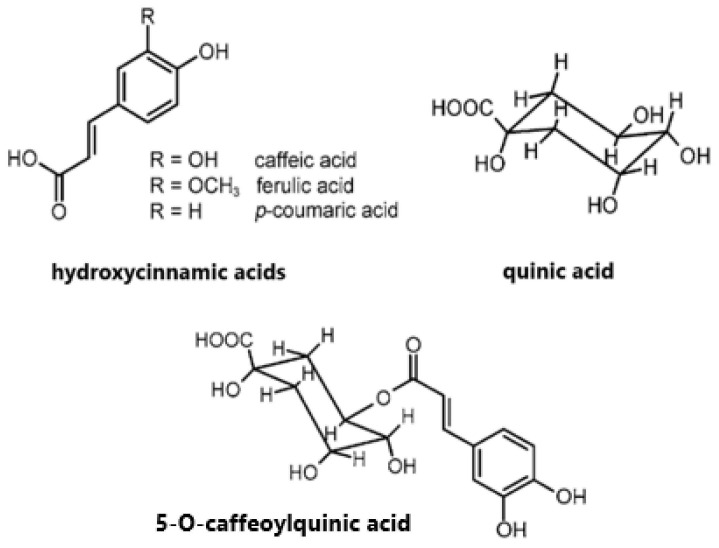
Chemical structures of main chlorogenic acids in coffee.

Similarly to other phenolic compounds, chlorogenic acids are secondary plant products that are present to protect against environmental stress [37]. The term chlorogenic acids refers to a family of esters between hydroxycinnamic acids (ferulic acid, *p*-coumaric acid, caffeic acid) and quinic acid (Figure 1). The main subgroups of CGA found in green coffee beans are caffeoylquinic acids, dicaffeoylquinic acids, and feruloylquinic acids. They exist in multiple isomeric forms. The 5-O-caffeoylquinic acid (5-CQA) is the most abundant in green coffee beans and is often called just chlorogenic acid [6,38]. Its concentration is about 100 mg/g DM and represents 76–84% of the total content of CGAs [38]. The varied content of total chlorogenic acids and 5-CQA depends on the roasting conditions. The higher the roasting degree and temperature during this process, the lower the content of CGAs as they are decomposed with exposure to heat [38,39]. 

In general, about one-third of the ingested amount of chlorogenic acids through drinking coffee can be absorbed in the human gastrointestinal tract and metabolized in the stomach, intestine, liver, and kidney [38,40,41]. It was found that chlorogenic acid and other phenolic acids from coffee brews show a very high correlation with microbiome components and that this relationship was highly reproducible across different populations [41]. Coffee consumption may also affect the absorption, distribution, and metabolism of some drugs [42]. For example, the findings by Kim et al. suggest that the intake of coffee can increase the absorption of aspirin by modifying the gut microbiome [43]. On the other hand, the addition of coffee to phenothiazine drugs forms an insoluble precipitant and decreases the absorption of neuroleptic medicaments [42].

**Figure 2:**
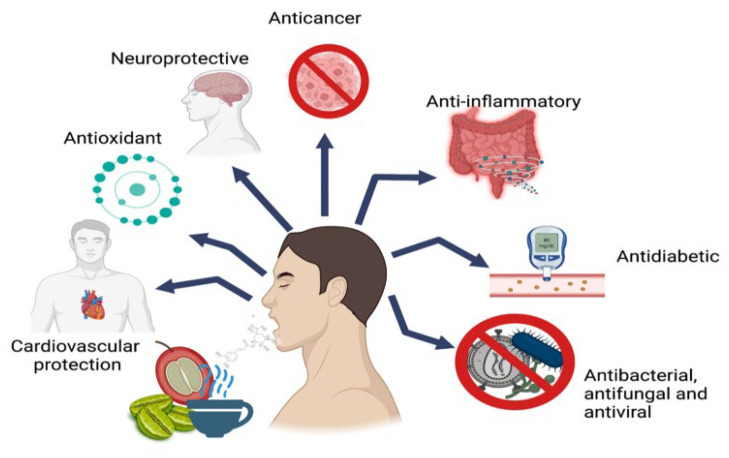
Main biological activities attributed to CGAs. Reprinted under the terms of the CC BY license from reference [6]. Copyright MDPI 2022.

Considering the results from animal and human studies, it was concluded that the dietary consumption of coffee CGAs is associated, to a certain extent, with a delay in the development of various chronic diseases and age-related disorders [35,40,44]. Chlorogenic acids exhibit multidimensional functions, including antioxidant and anti-inflammatory activities [45,46], anticancer [47,48], antibacterial [49], antiviral effects [50], and neurodegenerative properties [51,52]. CGAs have been proven to have a good protective effect on liver and kidney injury [53] and type-2 diabetes [54]. The recent advances of CGAs in terms of their cardiovascular-preserving effects were summarized by Lin et al. [55]. The main biological activities attributed to CGAs are present in Figure 2.

This wide range of biological activities of chlorogenic acid is mostly attributed to its antioxidant activity, which donates its five phenolic hydroxyl groups to free radicals. This process mitigates lipid peroxidation and DNA damage and contributes to the overall protection of tissues against oxidative stress. It has also been shown to elevate the expression of antioxidant enzymes [45]. However, as with many phenolic compounds, chlorogenic acids can also act as pro-oxidants, generating reactive oxygen species through the Fenton reaction. It depends on their concentration, free transition, the presence of metal ions, and their redox status [56,57]. 

The fortification of foods with coffee CGAs has the potential to improve food functionalities [6,58,59]. CGAs are highly stable in yogurt and soymilk, with improved flavor, texture, and other sensory attributes. Shelf-life studies revealed the sustained viability of dairy and kefir cultures throughout 21 days of storage [58]. The presence of CGAs also increased the antioxidant activity of the final products [58,59]. 

Preparations containing chlorogenic acids are used in cosmetics in the form of creams, face masks, and serums. They counteract the photo-aging of the skin, show depigmenting properties, and are recommended in the regeneration of acne, seborrheic, and atopic skin [18,19,20]. 

## 3. Extraction of Chlorogenic Acids from SCGs

The extraction of chlorogenic acids from spent coffee grounds has been a subject of significant interest as these compounds have potential applications in functional foods, dietary supplements, and cosmetic products. Some examples of the commercialization of such products were presented by Arias et al. [11]. Klingel et al. reviewed novel coffee products in the food sector (with the use of coffee by-products) and their current legal classification in the European Union [60].

Obtained extracts from spent coffee grounds are often characterized in terms of their total polyphenol content (TPC) using the Folin–Ciocalteu (FC) assay, as other phenolics are also extracted under a given condition. The value of TPC is usually expressed as the gallic acid equivalent per gram of a sample (GAE/g). However, the FC reagent is not specific only for phenolic compounds, and it can simultaneously oxidize several other compounds (e.g., sugars, ascorbic acid, or metal ions), giving elevated apparent TPC values [61]. The extraction yields are also expressed in terms of related antioxidant activities using different in vitro chemical assays. Some of these spectrophotometric methods are concerned with electron or radical screening, whereas others are focused on their reducing ability [62]. Additionally, these assays use different chromogenic reagents and different conditions for measurement. Thus, the relations between the content of CGAs, the TPC value, and the antioxidant capacity of an obtained extract are not always positive and can be obtained. Advanced chromatographic techniques (e.g., HPLC-MS/MS, HPLC-ESI-MS-MS) can help obtain accurate insights into the composition.

Several parameters, like solvent type, extraction temperature and time, and the liquid–solid ratio, influence the efficiency of extraction of CGAs from spent coffee grounds. In several cases, the optimum values of these parameters are determined using a response surface methodology (RSM). Generally, a longer time is required when extraction is carried out at room temperature. Heating enhances the extraction yield, but a long period of extraction at elevated temperatures may cause the undesirable degradation of chlorogenic acids [63].

One should also keep in mind that the obtained extracts using polar solvents, except CGAs and other phenolics, may also contain other biocompounds, such as caffeine, among others. Caffeine also has the potential to neutralize hydroxyl radicals and superoxide anions and contribute to the overall antioxidant activity of a given sample [64,65]. 

### 3.1. Conventional Extraction Methods

Conventional solid–liquid extraction methods, such as maceration, extraction in the Soxhlet apparatus, and under reflux, are often used due to their simplicity, efficiency, and wide range of applications. CGAs can be extracted by polar solvents such as methanol, ethanol, and acetone or by mixing them with water. Although methanol exhibits high polarity and high extraction yields, ethanol is recommended as more suitable for food applications. The mixture of alcohol with water is more effective than pure alcohol because water helps the diffusion of extractable components through plant tissues [63,66,67]. As was studied by Zengin et al., the 5-CQA and total phenolic contents in the SCG extracts were decreased in the following order for methanolic aqueous solvents: 50% methanol > 100% methanol > water [67]. The same order was found for the DPPH (2,2-diphenyl-1-picrylhydrazyl) and ABTS (2,2′-azino-bis(3-ethylbenzothiazoline-6-sulphonic acid) assays used to evaluate the quench of free radicals. However, in CUPRAC (cupric-reducing antioxidant capacity) and FRAP (ferric-reducing antioxidant power), which both evaluate the reducing power of a sample, a higher value was obtained for 100% methanol than for water.

The optimization of the extraction conditions from SCGs using the desirability approach was presented by Gigliobianco et al. [68]. The total phenolic content of 61.49 mg GAE/g was determined in the extract produced from 1 g in 20 mL of water at 80 °C for 30 min. Phenolic acids in several SCG samples from Vietnamese coffees were extracted in a shaker with pure methanol for 24 h at room temperature [69]. Chlorogenic acid predominated over the other phenolic acids in all the samples, with a wide concentration range of 0.83 to 7.66 mg/g and a median of 3.07 mg/g. The TPC values of these samples ranged from 109.4 to 181.3 mg GAE/g of the extract. The CGA content in the range of 0.02–4.8 mg/g was determined in SCGs from different coffee varieties purchased from local markets in Spain when extraction was conducted with the ethanol–water mixture (25:75) at 60 °C for 15 min [70].

Bouhzam et al. compared the efficiency of ethanol–water and acetone–water mixtures with different proportions for the recovery of chlorogenic acid [71]. A 0.7 g sample of SCG was mixed with 4 mL of the studied solution and stirred in a vortex shaker for 1 min at room temperature. The results revealed that water (0.79 mg/g) and 20% acetone (0.83 mg/g) were slightly better than 20% ethanol (0.74 mg/g). Extending the extraction time to 10 min increased the yield of CGA extraction to 0.99 mg/g using 20% acetone.

**Figure 3:**
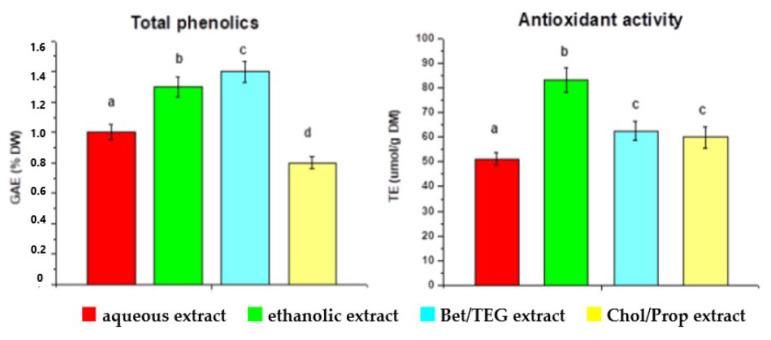
Total phenolic content (expressed in % GA of dry weight) and antioxidant activity in the DPPH assay (expressed in Trolox equivalent μmol/g DW) in different extracts. The letters above the error bars stand for statistically significant differences between groups. Reprinted under the terms of the CC BY license from reference [73]. MDPI 2023.

**Figure 4:**
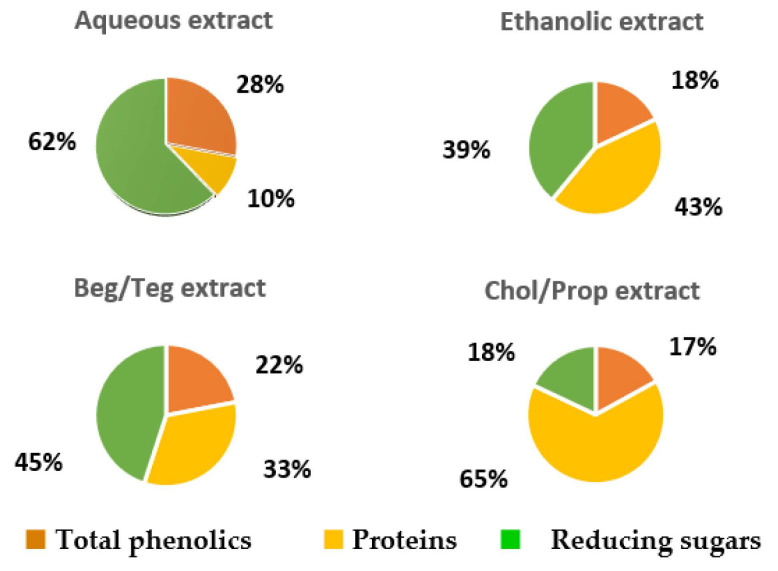
Pie charts representing the composition of phenolics, proteins, and reducing sugars in different extracts. For each extract, 100% is the sum of three components. Bet/TEG—betaine/triethylene glycol; Chol/Prop—choline chloride/1,2-propanediol. Reprinted under the terms of CC BY license from reference [73]. MDPI 2023.

The types of green solvents are represented by deep eutectic solvents (DESs) and natural deep eutectic solvents (NADEs) [72]. The nature of their interactions with the extracted molecules is similar to only NADEs containing natural components. They are analogs of ionic liquids containing hydrogen bond donor and hydrogen bond acceptor molecules, creating strong bonds between them. The resulting eutectic mixture has a much lower melting point than the individual components, and they are more suitable for the extraction of the thermolabile compound. García-Roldán et al. evaluated the use of two NADESs, namely, betaine/triethylene glycol (Bet/TEG) and choline chloride/1,2-propanediol (Chol/Prop), both at a molar ratio of 1:2, as sustainable green solvents for the extraction of phenolics from SCGs [73]. The best extraction yield for the total phenolic content was obtained with a Chol/Prop mixture, followed by 60% ethanol (Figure 3). The results for the antioxidant activity measured by the DPPH assay showed that the Chol/Prop extract had lower activity than the ethanolic extract, which could be due to differences in the profiles of phenolic compounds or due to other non-polyphenols that contributed to the antioxidant activity (e.g., melanoidins). In addition, each extractant showed a different selectivity for other main SCG components. Figure 4 presents the composition of phenolics, proteins, and reducing sugars for each extract (100% is the sum of these three components). The aqueous extract contained the highest fraction of reducing sugars, followed by Beg/TEG, ethanolic, and Chol/Prop extracts. Bet/TEG and aqueous extracts had similar fractions of phenolic compounds, much greater than others. According to the authors, the Chol/Prop extract could be useful as a food ingredient due to its low sugar content and high protein content. Please note that only three main factions of the many compounds found in SCGs were taken into consideration. SCGs also contain lignin, hemicellulose, carbohydrates, fatty acids, and caffeine in different quantities, which may be extracted under given conditions [9,10,11,12].

Conventional liquid extraction is often assisted with ultrasounds or microwave action, which facilitates the release of extractable compounds. In ultrasound-assisted extraction (UAE), the propagation of sound waves creates acoustic cavitation, and the collapse of cavitation bubbles mechanically disrupts the plant cell wall [74]. The application of UAE decreases the volume of a used solvent and extraction time. In microwave-assisted extraction (MAE), the process occurs due to changes in the cell structure caused by electromagnetic waves. [75]. The extraction efficiency of chlorogenic acids (quantified as 5-O-caffeoylquinic acid) using water decreased in the following order: UAE at room temperature for 1 min (1.15 mg/g) > UAE at 50 °C (1.02 mg/g) > only water vortex-shaken (0.82 mg/g) [76]. The results obtained also revealed significant differences between the two samples of SCG brands. For one of them, the content of 5-CQA was 1.15 mg/g, as mentioned earlier, and for another SCG sample under the same extraction conditions, 5-CQA was three times smaller (0.345 mg/g). This was attributed to their origin, roasting degree, and procedure for the first coffee infusion preparation. 

Yoo et al. proposed the UAE method and deep eutectic solvent consisting of 1,6-hexanediol/choline chloride (molar ratio 7:1) to recover phenolic compounds from SCGs [77]. The extraction was conducted at 60 °C for 10 min, yielding a TPC value of 17.0 mg GAE/g. Adsorption chromatography was applied to recover phenolics from DES extracts with more than 90%. There is the possibility of recovering phenolic compounds from DESs extracted in different ways, although this additional process lengthens the procedure. Several used DESs come from natural sources and are generally recognized as safe; thus, they can be incorporated into the formulation of new products [78].

The polarity of solvents plays an important role in microwave-assisted extraction, and solvents that possess higher dielectric factors and dissipation are advisable [14,79]. As a result, water, methanol, or ethanol are more suitable than chloroform or hexane. Coelho et al., applying the design of experiments for the MAE of phenolic acids, identified that the content of ethanol in its mixture with water and the ratio of solvent to SGG mass are the most significant parameters [80]. Under the optimum extraction conditions (69% EtOH, solvent/SCG ratio of 16.7), the total phenolic content was 117.7 mg GAE/g, and the antioxidant activity in the DPPH assay was equal to 143.8 μmol TE/g. The temperature and time of extraction can also influence the extraction yields. High temperatures and long heating times are often used to promote the extraction process, but microwave radiation combined with high temperatures may cause the degradation of phenolic compounds. Pettito et al. analyzed the extraction kinetic of SCG using MAE with 54% ethanol [81]. The effects of the initial thermal ramp (i.e., heating time once extraction temperature was fixed) on extraction yields were investigated when the temperature was kept under controlled conditions. These investigations revealed that 10 min of heating time at 150 ºC provided the highest concentration of polyphenols. According to them, this behavior implies that even if polyphenol degradation occurred, the enhancement of extraction kinetic due to temperature increase would be more effective than degradation reactions.

### 3.2. Advanced Extraction Methods

Advanced extraction techniques using elevated pressure and temperature have advantages over conventional methods as they are generally faster with minimum solvent consumption [82]. They use different solvents for extraction, such as pressurized liquids, supercritical fluids, or enzymes, to enhance the efficiency of the isolation of CGAs from spent coffee grounds. In pressurized liquid extraction (PLE), high pressure (usually up to 600 MPa) is used for short periods (5–10 min) to treat a sample [83]. High hydrostatic pressure-assisted extraction (HHPE), performed in the range of 100–900 MPa, can be performed at room temperature and prevent the degradation of heat-sensitive compounds [84]. The study by Wu et al. employed the hydrothermal method to extract components of SCGs [85]. Using high temperature (150–200 °C) and high-pressure water treatment for 3 h allowed a TCP value equal to 9.44 ± 0.90 mg GAE/g to be obtained.

Okur et al. compared HHPE and UAE techniques with SLE using 80% MeOH for 15 min according to the total phenolic content and antioxidant activity [86]. After sonication, the extract had a higher chlorogenic acid content (85.0 ± 0.6 mg/kg FM) compared to HHPE (81.2 ± 1.1 mg/kg FM) and classical liquid extraction in a water bath at 50 °C for 30 min (24.0 ± 0.3 mg/kg FM). Both UAE and HHPE gave higher TPC values and antioxidant activity in the DPPH assay (9.5 GAE/100 g) compared to the SLE method. 

Among the green technologies, extraction with carbon dioxide in liquid and supercritical conditions is suitable for thermally labile substances. CO_2_ is a green, nonflammable solvent that can be easily removed from the final product by changing pressure. It exists in a supercritical state under the conditions of temperature and pressure above the critical point and possesses hybrid properties between gas and liquid. In supercritical fluid extraction (SFE) with carbon dioxide for polar compounds, such as polyphenols, a small addition of polar co-solvents (mainly methanol or ethanol) can be used [87,88,89,90]. Romano et al. compared the efficiency of extraction with CO_2_ in liquid and supercritical conditions, both with and without the addition of ethanol as a co-solvent, to recover bioactive compounds from SCG espresso [91]. Other phenolic acids, some fatty acids, and caffeine were also identified in the extracts. In the extracts without a co-solvent, CGA was not detected. The addition of 5% ethanol during extraction led to values of 4.70 mg/100 g oil and 9.08 mg/100 g oil in the LE and SFE methods, respectively, due to ethanol polarity and its affinity for phenolics. This trend was also observed for the total content of phenolics. 

The recovery of CGAs, as well as a phenolic fraction from spent coffee grounds, have been used in a range of extraction methods. The recently published procedures are shown in Table 1, where a wide range of reported values can be observed. This fact can be attributed to the differences in raw materials (Robusta beans and lightly roasted coffee are richer in chlorogenic acids), the extraction method, and the type of solvent. Each method offers some advantages and disadvantages. Moreover, the results are often expressed in different units, making it difficult to compare them.

Generally, advanced extraction methods allow for more efficient recovery from different coffee by-products. However, the recovery of CGAs and phenolic compounds in different coffee by-products is highly dependent on temperature [4,10,76]. Applying high-temperature, high-frequency ultrasounds, and high-power microwaves enables the faster destruction of matrix cellular structures but may lead to the hydrolysis and oxidation of numerous phenolic compounds [82,92]. Thus, the appropriate extraction conditions have to be carefully selected. Further processing of the extracted CGAs has been evaluated to protect them from oxidation and preserve their properties for a longer time. The encapsulation of phenolic compounds and antioxidants obtained from SCG extracts has been proposed to solve this problem using different isolated and combined wall materials [93,94].

## 4. Conclusions

Increased concern over the safety of synthetic antioxidants has led to growing attention to replacing them with effective and economical natural compounds that exhibit antioxidant activity. Natural antioxidants can be found in the leaves, fruits, grains, and roots of plants and have been used as medicine for ages. Agricultural by-products are another source of these compounds. These forms of waste contain several bioactive compounds, and their valorization leads to a circular economy. Spent coffee grounds are still rich in phenolic compounds, can be isolated through different extraction processes, and have diverse potential applications. This overview presents the recently proposed procedures for extracting and recovering chlorogenic acids together with phenolic fractions from spent coffee grounds. SCGs represent the most abundant waste generated in coffee beverage preparation and instant coffee production. Interested readers could find earlier contributions in the review papers [10,14,27].

Several extraction procedures using different solvents and techniques for the recovery of CGAs have been proposed, including organic solvents, mixing them with water, and deep eutectic solvents. Their action is supported by ultrasounds or microwaves to facilitate the release of chlorogenic acids. Advanced extraction technologies such as pressurized liquid extraction, high hydrostatic pressure-assisted extraction, and supercritical fluid extraction are generally faster and enhance the efficiency of extraction yields. The extraction efficiency of a given group of compounds from natural sources depends on the raw materials, such as genetic variation, bean maturation, agricultural practices, and processing methods. Another group of parameters that influence this process are extraction conditions, like solvent type, temperature, and extraction time. Solvent selection and temperature control seem to be significant factors due to the thermolabile nature of CGAs. In terms of SLE, the application of natural deep eutectic solvents is a promising method as it reduces the use of organic solvents, operating at mild temperature conditions with sustainable and natural compounds. However, more research is needed during optimization to obtain solvent-free CGA extracts.

Another aspect of isolating chlorogenic acids is the influence of spent coffee grounds’ storage time on extraction efficiency. Bouhzam et al. found a significant decrease in the concentration of 5-CQA by 82% after an eight-month storage period, and it was recommended to limit the storage time to less than 4 months to maintain its level as close as possible to the initial levels [71]. Quality control after the extraction process and the stability of CGAs should also be considered. Spent coffee grounds contain several biocompounds, and the majority of publications have only investigated chlorogenic acids, caffeine levels, and total polyphenol contents. Recently, Angleoni et al. proposed a new analytical method for the quantification of 30 bioactive molecules in SCGs using UAE with 70% ethanol for extraction and HPLC-MS/MS [95]. Except for these compounds, other substances, e.g., melanoidins generated in the Maillard reaction between proteins and sugars during the roasting of coffee beans, have also been identified [8,96,97]. Melanoidins are responsible for the color and aroma of heat-processed foods and also exhibit antioxidant activity [98]. However, such thermal processes can also lead to the generation of some toxic compounds like aromatic polycyclic hydrocarbons and various mycotoxins [99,100]. Thus, the roasting conditions of coffee should be controlled to avoid the formation of these toxigenic compounds, and the control of the obtained products and the further use of by-products is desirable.

The proposed procedures for the extraction of chlorogenic acids have been mostly applied at a laboratory scale, and only a few works have investigated larger-scale solvent extraction experiments [101,102]. The choice of an appropriate extraction method for industrial-scale applications should consider the design of much larger-scale extraction equipment, the control of production costs, processing time, energy, and the environmental impact of industrial production [2,11,103]. Peluso explored the multifaceted opportunities and economic benefits that originate from the utilization of coffee by-products and the diverse applications that contribute to their economic significance [104].

## Figures and Tables

**Table 1:** Recently published procedures for the extraction of chlorogenic acids from SCGs.

Extraction Method	Extraction Conditions	CGAs Content mg/g	TPC (mg GAE/g)	Ref.
SLE	water, 1 g/20 mL, 80 °C, 30 min	-	61.49 ± 1.36	[68]
SLE	0.7 g/4 mL, room temp., 1 min water 20% EtOH 40% EtOH 20% acetone 40% acetone	0.78 0.74 0.35 0.83 0.69	3.83 3.98 3.93 4.40 4.37	[71]
SLE	NADES, 1 g/15 mL, 65 °C, 150 min Bet/TEG Chol/Prop 60% EtOH, 1 g/8 mL, 60 °C, 2 h water, 1 g/8 mL, 100 °C, 1 h	-	1.42 ^1^ 0.78 ^1^ 1.26 ^1^ 0.97 ^1^	[73]
UAE	10 g/50 mL, 20 °C, 120 min water 100% MeOH 50% MeOH 70% EtOH	4.66 ± 0.25 9.79 ± 0.82 10.61 ± 0.90 8.62 ± 0.76	56.86 ± 0.16 63.25 ± 0.10 93.26 ± 0.14 93.55 ± 0.65	[67]
UAE	water, 0.7 g/4 mL, 1 min, room temp. 50 °C	1.15 1.02	-	[76]
UAE	DES (1,6-hexanediol/choline chloride, 7:1), 100 mg/2.6 mL, 60 °C, 10 min	-	17.0 ± 0.2	[77]
MAE	70% EtOH, 1 g/15 mL, 6 min, 75 °C	-	117.7 ± 6.1	[79]
SLE UAE HHPE	80% MeOH, 50 °C, 30 min 80% MeOH, 15 min 80% MeOH, 15 min	24.0 ± 0.3 ^2^ 85.0 ± 0.62 ^2^ 81.2 ± 1.1 ^2^	6.40 ± 0.18 9.51 ± 0.06 9.42 ± 0.10	[86]
LE-CO_2_ SFE-CO_2_	10 mL/min flow rate, 30 MPa, 1 h 20 °C + 5% EtOH 60 °C + 5% EtOH	1.41 ± 0.16 ^2^ - 2.01 ± 0.06 ^2^	692.75 ± 55.00 ^3^ 857.25 ± 37.00 ^3^ 419.50 ± 66.00 ^3^ 969.75 ± 35.00 ^3^	[91]

^1^ Expressed in GAE % of dry weight; ^2^ expressed in mg/kg FM; and ^3^ expressed in mg/100 g oil. SLE—solvent liquid extraction; UAE—ultrasound-assisted extraction; MAE—microwave-assisted extraction; HHPE—high hydrostatic pressure-assisted extraction; SFE—supercritical fluid extraction; MeOH—methanol; EtOH—ethanol; DES—deep eutectic solvent.

## Data Availability

No new data were created or analyzed in this study.
